# Selumetinib in Adult Neurofibromatosis 1 with Plexiform Neurofibroma

**DOI:** 10.3390/ph18071039

**Published:** 2025-07-13

**Authors:** Carlen A. Yuen, Eleanor Chu, Ryan O’Connell, Bryan K. Sun, Raj Vyas, Michelle Zheng, Emma Elliott, Changrui Xiao

**Affiliations:** 1 Department of Neurology, Division of Neuro-oncology, University of California Irvine, Irvine, CA 92697, USA; 2 Chao Family Comprehensive Cancer Center, University of California Irvine, Irvine, CA 92697, USA; 3 Department of Radiology, University of California Irvine, Irvine, CA 92697, USA; 4 Department of Pathology, University of California Irvine, Irvine, CA 92697, USA; 5 Department of Dermatology, University of California Irvine, Irvine, CA 92697, USA; 6 Department of Plastic & Aesthetic Surgery, University of California Irvine, Irvine, CA 92697, USA; 7 UC Irvine Charlie Dunlop School of Biological Sciences, University of California Irvine, Irvine, CA 92697, USA; 8 Department of Psychology, University of California San Diego, La Jolla, CA 92093, USA; 9 Department of Neurology, University of California Irvine, Irvine, CA 92697, USA

**Keywords:** neurofibromatosis 1, NF1, selumetinib, adult, plexiform neurofibroma, targeted therapy

## Abstract

**Background/Objectives:** Neurofibromatosis Type 1 (NF1) plexiform neurofibroma (PN) can cause morbidity, including disfigurement that can negatively impact social functioning. Historically, the mainstay treatment is surgical resection. However, complete resection is often prohibitive due to multiple nerve involvement. Moreover, post-operative recurrence is common. MEK inhibitors, including selumetinib and mirdametinib, have recently changed the treatment paradigm for these tumors. In 2020, selumetinib was FDA-approved for pediatric NF1 patients with inoperable symptomatic PNs, but selumetinib remains under investigation for their adult counterparts. In 2025, mirdametinib was FDA-approved for use in adults with symptomatic incompletely resectable NF1 PNs. Lower partial response rates have been reported with mirdametinib compared to selumetinib, but direct comparative analyses have not been conducted to establish the superiority of one agent over the other. **Results:** We present a case of a 38-year-old male with a right facial PN successfully treated with selumetinib, resulting in a 16.77% tumor volumetric reduction over 7 months. Selumetinib was well tolerated in our patient, with an asymptomatic Grade 3 CPK elevation that subsequently improved with a dose reduction. **Conclusion:** Our case adds to the growing body of evidence suggesting that selumetinib is effective and well tolerated in adult patients with NF1-associated PNs.

## 1. Introduction

The Neurofibromin 1 (MIM# 613113, *NF1*) gene encodes neurofibromin, a tumor suppressor protein that negatively regulates the Ras signaling pathway [1,2]. Neurofibromin converts active guanosine triphosphate-bound RAS to an inactive GDP-bound RAS [3]. As such, heterozygous loss-of-function and inactivating mutations within *NF1* upregulate and constitutively activate the MAPK signaling pathway, leading to tumorigenesis [3,4,5,6,7,8,9].

Pathogenic germline variants occur in approximately half of all NF1 cases, giving rise to Neurofibromatosis type 1 (NF1) syndrome (MIM# 162200, NF1) [10,11]. NF1 is an autosomal-dominant inherited tumor predisposition syndrome that is characterized by a broad spectrum of diagnostic findings, including café-au-lait spots, intertriginous freckling, bone dysplasia, and tumors [10,12,13,14]. This condition is clinically heterogeneous, with varying levels of multisystem involvement–even within the same family [15]. Approximately half of these patients develop plexiform neurofibromas (PNs). PNs are tumors comprising of Schwann cells, fibroblasts, and hematopoietic and mast cells, among others [16]. PNs can develop anywhere from the nerve root to the distal peripheral nerve, and may cause morbidity and disfigurement for NF1 patients [2,9,15,17,18,19]. Mortality may be increased in cases of transformation to malignant peripheral nerve sheath tumor, airway compression, or spinal cord impingement [10,20,21,22]. Associated symptoms depend on the tumor location and may have significant impacts on physical functioning, including pain, motor, vision, bowel, and bladder impairment [10,19,23]. PNs causing disfigurement can significantly impact social functioning and limit activity engagement and participation [24,25]. Limited studies address the negative social impact associated with this syndrome. The stigma associated with the appearance of these tumors can bear emotional weight on the lives of these patients [26], including those with facial deformity.

NF1 syndrome may be diagnosed clinically or genetically, with PN as a diagnostic criterion [27]. PNs are diagnosed on MRI and surgical resection is the mainstay treatment for these tumors [2,12,17,28,29,30]. However, multiple nerve involvement, soft tissue invasion, and vascular injury often prohibit complete tumor resection [2,12,17,28,29,31]. Furthermore, 40–50% of PNs recur following surgery [12,29,32,33]. Accordingly, there has been an unmet need for the treatment of these tumors. Trials investigating therapies for NF1 PNs have been primarily focused on the pediatric patient population, wherein PNs exhibit rapid growth during childhood and in the setting of hormonal changes [34,35,36,37,38,39]. In stark contrast, adult NF1 PNs exhibit an insidious growth pattern [35,37,39]. Agents targeting multiple pathways, including pegylated interferon alfa-2b, pirfenidone, tipifarnib, sirolimus, and imatinib, have shown marginal success with limited reductions in tumor volume [40,41,42,43,44,45]. However, molecular advances have now led to the emergence of targeted therapies.

MEK inhibitors (MEKi)s aimed at the MEK gene hinder the MAPK signaling cascade [2]. Selumetinib is a potent allosteric kinase MEK 1/2 inhibitor that impedes ERK1/2 phosphorylation, consequently reducing cell proliferation, survival, and differentiation [46,47,48]. Selumetinib can induce durable responses with no apparent activation of resistance pathways, including the AKT signaling pathway [37]. The pivotal National Cancer Institute (NCI) Phase 1/2 SPRINT trial (NCT01362803) investigating the use of selumetinib in children with symptomatic inoperable NF1 PNs demonstrated partial response rates (PR = tumor volume decrease of 20% or greater for 4 or more weeks) of 68–75%, many of whom maintained durable responses lasting more than one year, and a median progression-free survival of 7 years (Table 1) [17,49]. Accordingly, selumetinib was granted FDA approval for the treatment of NF1 pediatric patients with inoperable PNs based on the success of this trial [50,51]. In a subsequent Phase II study conducted by Kim et al., 87% (26/30) of adult NF1 PN patients treated with selumetinib 50 mg twice daily (BID) achieved PR [52]. All participants who completed 26 cycles achieved PR [52]. In another Phase 2 study (NCT02407405) investigating the use of selumetinib in adult patients with inoperable, progressive, or symptomatic PNs, the overall response rate (ORR) was 63.6% [37]. The safety profiles amongst these trials are comparable, with reported adverse events (AEs) of rash, elevated creatine phosphokinase (CPK) or alanine transaminase, dry skin, and pruritus [17,49,52]. Selumetinib has yet to gain regulatory approval for use in NF1 PN adult patients, but it remains under investigation. The randomized, placebo-controlled Phase 3 KOMET (NCT04924608) and NCI Phase I (NCT02407405) trials are currently underway to assess the efficacy and safety of selumetinib in adult NF1 patients with inoperable PNs. Interim results for the KOMET study are promising, with an ORR of 20% and a 33.9% reduction in tumor volume in participants who completed 16 cycles of selumetinib 25 mg/m^2^ BID [53]. Notably, even participants who completed only 4 months of selumetinib demonstrated a response to therapy [53]. More recently, in February 2025, mirdametinib was FDA-approved for use for NF1 adults (and children) with symptomatic PNs who are not amenable to complete resection, based on the success of the ReNeu trial [54]. Interestingly, the PR of 42% associated with mirdametinib is lower than the ORR associated with selumetinib for NF1 PN adults (87%) [9,52]. However, the ReNeu trial was not designed to determine the maximal tolerated dose, and higher dosing may have positively influenced the therapeutic response [9]. Further, mirdametinib provides the additional benefit of a liquid formulation to accommodate NF1 head and facial PN patients with dysphagia [25].

Herein, we present the case of a 38-year-old male with NF1 and a right facial PN who was successfully treated with selumetinib, with a tumor volumetric response of −16.77% following 7 months of therapy. Our case adds to the growing body of evidence supporting the use of selumetinib in adults with NF1 PNs.

## 2. Case Presentation

**Figure 1:**
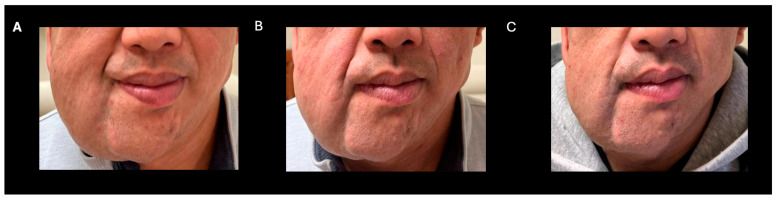
Clinical improvement after selumetinib (**A**) baseline (**B**) 3 months post-selumetinib (**C**) 7 months post-selumetinib.

**Figure 2:**
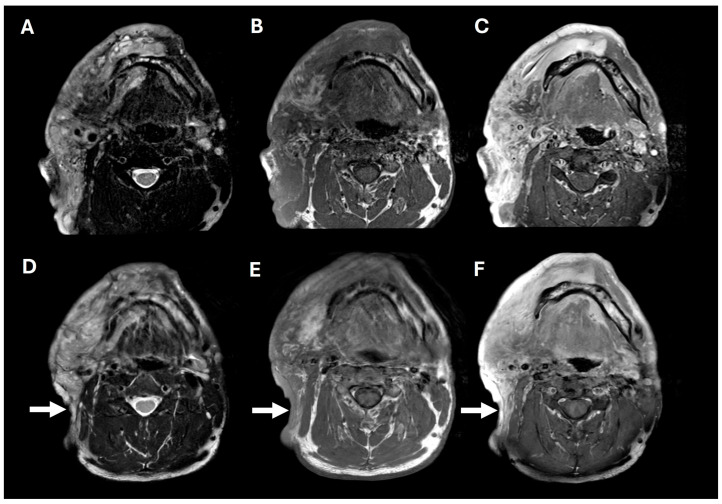
Pre- and post-operative brain MRIs. Pre-operative T2, T1, and T1+contrast imaging (**A**–**C**), showing a large, T2-hyperintense, enhancing, predominantly right facial mass. Post-operative T2, T1, and T1+contrast imaging (**D**–**F**), showing posterior tumor debulking (arrows).

**Figure 3:**
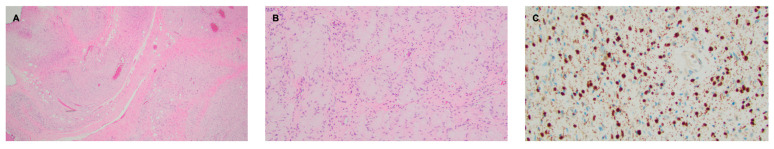
Right facial plexiform neurofibroma. (**A**) H&E stain, 2× objective, highlighting a diffusely infiltrative proliferation of spindled to ovoid cells with loose collagenous stroma. A vague bundled and tortuous pattern is visible at this power. (**B**) H&E stain, 10× objective, demonstrating an area featuring abundant pesudo-Meissner corpuscles, a feature frequently encountered in diffuse and plexiform neurofibromas. (**C**) S100 stain, 20× objective, showing nuclear and granular cytoplasmic staining, as compared to the diffuse staining seen in schwannomas.

**Figure 4:**
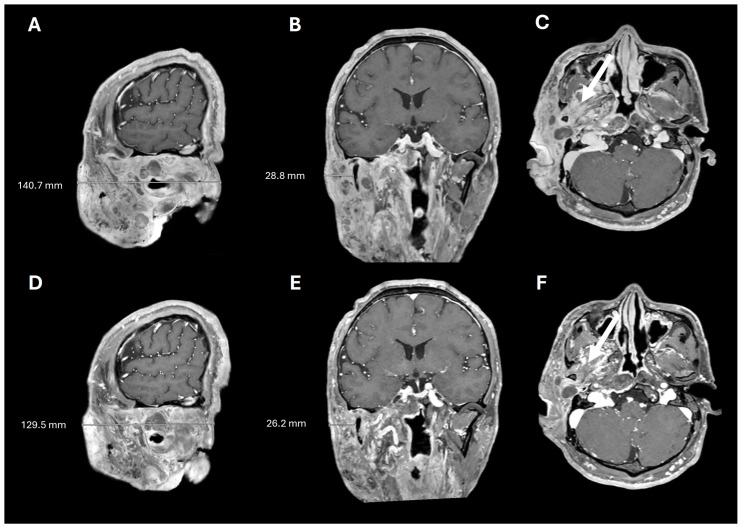
Pre- and post-selumetinib brain MRI. T1+contrast imaging before (**A**–**C**) and after (**D**–**F**) 7 months of selumetinib, showing decreased tumor burden and decreased tumor infiltration into the masticator space (arrows).

A 38-year-old man with genetically confirmed NF1 and a large conglomerate R facial PN, with status post debulking at age 8, presented to our institution for further management of his disfiguring PN. His family history was positive for NF1 in his mother and daughter. Genetic testing revealed a heterozygous pathogenic truncating variant in exon 38 of NF1: c.5438C>A and p.Sesr1813Ter. No reportable variants were found in SPRED1. Neurological examination showed a large right facial mass (Figure 1A), >6 café au lait macules—all >15 mm in size, axillary freckling, and numerous cutaneous neurofibromas. A brain MRI demonstrated a right predominantly facial PN (Figure 2A–C). Following posterior auricular tumor debulking, a brain MRI showed an interval reduction in the tumor size posterior to his ear (Figure 2D–F). Pathological examination showed positive S100, SOX-10, and CD34, and negative SMA, consistent with neurofibroma (Figure 3). Ki-67 was less than 1%. Molecular analysis identified a pathogenic variant (NM_000267.3: c.5438C>A, p.Ser1813Ter) in the heterozygous state in *NF1*. At the time of diagnosis, there were no FDA-approved agents for adult NF1 PNs. On the basis of the higher reported ORR in selumetinib compared to mirdametinib, selumetinib was the targeted agent selected for our patient. However, no head-to-head clinical trials have directly compared mirametinib and selumetinib. He was administered 25 mg/m^2^ (40 mg BID) selumetinib, which was complicated by a facial acneiform rash and asymptomatic elevated Grade 3 CPK after 1 month. His rash resolved with topical adapalene 0.1%/benzoyl peroxide 2.5% gel, and his CPK improved to Grade 1 with a dose reduction in selumetinib to 35 mg BID. At 7-month follow-up, a reduction in tumor burden was noted clinically (Figure 1A–C), with a corresponding reduction in tumor volume radiographically (Figure 4A–F). On his brain MRI, the pre-selumetinib tumor volume was 284.65 cm^3^ and the post-selumetinib tumor volume was 236.92 cm^3^, showing a 16.77% reduction in tumor volume. Given the infiltrative nature of the tumor, volume was calculated using an ellipsoid volume formula of 0.5 × length × width × height. At time of publication, he remains on selumetinib and endorses improvement in quality of life (QoL).

Ethical guidelines set out by the Declaration of Helsinki were followed in the preparation of this report, and the patient provided written consent.

## 3. Discussion

Plexiform neurofibromas may cause morbidity and disfigurement in adult NF1 patients [2,9,15,17,18,19]. Studies addressing the negative social impact of facial PNs on these patients are limited. Facial disfigurement can significantly affect social functioning for these patients and hinder participation in activities, leading to a poor QoL [24,25,26,56]. For these reasons, our patient sought further management to reduce his right facial tumor burden. However, NF1 PNs present surgical challenges due to their infiltrative nature, and effective therapy remains a treatment gap in neuro-oncology practice to date [28,38,57]. In particular, facial PNs are arduous due to multiple nerve involvement, soft tissue invasion, and vascular injury, which highlights the need for alternative therapies [28,57,58].

MEKis are a promising therapy to fulfill the treatment gap for adults with NF1 PNs. Recently, mirdametinib was FDA approved for this indication on the basis of the Phase 2 single-arm ReNeu trial [2,54], four months after our patient initiated treatment on selumetinib. However, of the adult NF1 PN participants treated with mirdametinib in this trial, the results were modest compared to results with selumetinib (PR = 42% vs. PR = 87%, respectively) [9,52]. At time of publication, selumetinib was regulatory approved for use in pediatric NF1 patients with symptomatic inoperable PNs, but was not approved for use in their adult counterparts [50]. An inverse correlation between age and NF1 PN growth rate has been proposed, with suggestions that pediatric NF1 PN patients may be more responsive to MEKis, compared to NF1 PN adults [9,34,35,36,37,59]. This increased sensitivity to MEKi has been attributed to higher PN growth rates during childhood compared to the insidious growth rate that is observed in adults with NF1 PN [2,9,35,39]. Despite this, our adult patient achieved significant tumor regression 30 years after his initial diagnosis, with a 16.77% tumor volumetric reduction following 7 months of selumetinib, and he reported improvement in QoL. Limited evidence suggests that neoadjuvant MEKi can provide benefit to permit surgery [60].

To our knowledge, only three other NF1 PN adults with facial PNs treated with MEKis have been reported; two patients were treated with selumetinib 50 mg/m^2^ BID, and one patient was treated with mirdametinib 2 mg/m^2^ (4 mg maximum) BID in a 3-week on/1-week off schedule [37,52]. The existing evidence suggests that tumor response can occur later in the treatment trajectory and initial non-responders may be later identified as delayed responders to MEKis [2,17,49]. Nevertheless, our patient was an initial responder and achieved a volumetric response after only 7 months of therapy, corroborating with reported results of some adult patients responding after only 6 cycles of therapy [52]. Kim et al. suggest that selumetinib pharmacokinetics may differ between Asian and Caucasian NF1 patients, with higher drug exposure in Asian individuals [52,61,62,63]. However, it remains unclear whether this factor contributed to our patient’s response to treatment.

This early response was attained on selumetinib 40 mg BID, with a dose reduction to selumetinib 35 mg BID due to asymptomatic Grade 3 elevated CPK. Other NF1 adult studies were designed with higher doses of selumetinib at 50 mg BID and 75 mg BID, and some assert that higher rates of response are associated with higher doses of selumetinib [37,52]. However, elevated Grade 3 CPK, though asymptomatic in our patient, prohibited a higher dose, and his CPK levels were sustained at Grade 1 following the dose reduction. Tumor regrowth was observed in a subset of patients requiring selumetinib dose reduction [17], but dose reduction did not negatively impact our patient’s response to treatment. Elevated CPK is a common adverse event observed in selumetinib and is customarily asymptomatic [17]. Avoidance of vigorous exercise can be recommended to prevent myositis in this scenario; however, our patient continued his regular exercise regimen without complication.

Our patient remains on selumetinib and may not have yet attained maximal volumetric response. The optimal duration of therapy with selumetinib to attain peak and sustained response remains unknown and is an area of future investigation [2,9]. Some assert that peak response is reached at 36–42 months [2]. Long-term use of selumetinib has acceptable rates of toxicity, and tumor recurrences have been reported in cases where selumetinib was discontinued [17,49,52,53,64]. For these reasons, he will continue with prolonged therapy, barring disease progression or any unacceptable treatment-related toxicities in the future. The significance of this study is highlighted by our patient’s early response to selumetinib, which may be due to differences in the pharmacokinetics of individuals of Asian origin and the limited number of reported cases of facial PNs treated with MEKis.

We acknowledge the limitations of this study. First, a single case is not generalizable to all NF1 adult patients with PNs. Second, we were unable to determine the durability and maximal volumetric response of selumetinib in our patient given the lack of long-term follow-up. Existing studies on MEKis report higher response rates with longer treatment durations [9,37,52,53,65]. Third, the pharmacokinetics of selumetinib was not analyzed in our patient, and it is unknown if the pharmacokinetics played a role in his tumor’s early response to treatment. Lastly, though our patient reported clinical improvement in his QoL following his tumor reduction, formal QoL assessments were not administered to our patient.

Efforts investigating other MEKis for the treatment of NF1 PNs, including trametinib and binimetinib, are ongoing [2,66]. Though single-agent selumetinib remains effective in our patient, a longer follow-up may uncover delayed resistance to therapy. Some studies suggest a resistance mechanism to selumetinib monotherapy with downstream activation of AKT [67,68,69]. However, data garnered from the KOMET study suggests otherwise [37]. If resistance mechanisms are uncovered, studies aimed at combinatorial therapy with MEKis and other agents should become areas of further investigation, including the addition of immunotherapy or another targeted agent within the MAPK pathway to MEKIs [2]. Alternatively, cabozantinib, a multi-targeted tyrosine kinase inhibitor that is not a direct MEKi, shows early promise for use in NF1 PN patients, with a PR of 42% in a Phase 2 study [55,70]. Cabozantinib is a small-molecule inhibitor of AXL, MET, and VEGFRs [70]. The mechanism by which cabozantinib exerts anti-tumor effects in PNs is not fully understood, but is postulated to be associated with downregulation of AXL, fibroblast regulation, and collagen production within the tumor microenvironment [55]. Lastly, insight into the ideal duration and need for continuous therapy can optimize MEKi treatment for these patients and determine the impact of a protracted course of MEKi therapy. Deepening our understanding of predictors of response, including single-cell RNA sequencing, may better target a subset of patients who can derive greater efficacy from MEKis [71]. Lastly, the PR rates between selumetinib and mirdametinib (87% vs. 42%) vary significantly [9,52]. The Phase II mirdametinib trial was not designed to determine the maximal tolerated dose for mirdametinib, and dosing above 2 mg/m^2^/dose may have potentially influenced therapeutic response [9]. Though this variance may potentially be attributed differences in trial design, no direct comparative analyses have been conducted [9]. Studies comparing different MEKis may reveal the superiority of one agent over the others.

## 4. Conclusions

Neurofibromatosis 1 plexiform neurofibromas can cause disfigurement and can negatively impact the social functioning of patients. Most NF1 PN investigations have been aimed at the pediatric population. Selumetinib, a MEK 1/2 inhibitor, achieved FDA approval for use in pediatric patients with symptomatic inoperable NF1 PNs, but it is currently under investigation for use in their adult counterparts. Mirdametinib was recently approved for use in adult patients with NF1 PNs, but it reports lower partial response rates compared to selumetinib. Comparative studies evaluating the superiority of one agent over the others have not been performed. Our case adds to the growing body of evidence suggesting that selumetinib is effective and well tolerated in NF1 PN adult patients. Prospective studies are indicated to evaluate the long-term efficacy, optimal dosing, and safety of selumetinib in adults with NF1-associated plexiform neurofibromas.

## Figures and Tables

**Table 1:** Clinical Trials investigating MEK inhibitors in NF1 patients with PNs. ORR = overall response rate; PR = partial response; BID = twice daily; CPK = creatine phosphokinase; ALT = alanine aminotransferase; PN = plexiform neurofibroma.

Author	Patient Population	MEK Inhibitor	Phase	Study Design	ORR (%)	ORR	Adverse Events
Chen et al. NCT04924608 KOMET [53]	adult	selumetinib 25 mg/m^2^ BID	III	placebo-controlled	20 (treatment arm); 5 (placebo) by cycle 16	REiNS	dermatitis acneiform (59%), increase blood creatine phosphokinase (45%), diarrhea (42%)
Dombi et al., NCT01362803 SPRINT [49]	pediatric	selumetinib 20–30 mg/m^2^ BID	I-II	single arm	68–75	PR (tumor volume decrease from baseline of at least 20% for at least 4 weeks)	acneiform rash, gastrointestinal effects, asymptomatic elevated CPK
Fisher et al. NCT02101736 [55]	adolescents and adults	cabozantinib 40 mg escalated to 60 mg after 2 cycles	II	single arm	42	PR (≥20% reduction in target lesion volume after 12 cycles of therapy)	gastrointestinal toxicity, hypothyroidism, fatigue, palmar plantar erythrodysesthesia
Gross et al. NCT02407405 [37]	adult	selumetinib 25 mg/m^2^ BID	II	placebo-controlled	63.6	REINS criteria	acneiform rash, (97%), elevated CPK (82%), dry skin (70%), pruritis (61%), increased ALT level (55%), limb edema (55%)
Kim et al. (adult and pediatric) [52]	adult and pediatric	selumetinib 50 mg BID	II	Single arm	91 (adult and pediatric); 87 (adult)	(≥20% volume reduction)	paronychia (14.7%) acneiform rash (14.5%) skin infection (14.0%)
Weiss et al. NCT02096471 ReNeu [9]	adolescents and adults	mirdametinib 2 mg/m^2^/dose (maximum dose = 4 mg twice a day)	II	single arm	42	≥ 20% reduction in tumor volume compared with baseline	acneiform rash (94.7%), fatigue (57.9%), nausea (52.6%)

## Data Availability

The data presented in this study are available on request from the corresponding author due to privacy and ethical restrictions.

## Abbreviations

The following abbreviations are used in this manuscript:

BID	twice daily
CPK	creatine phosphokinase
MAPK	mitogen-activated protein kinase
MEK	mitogen-activated protein kinase kinase
NF1	Neurofibromatosis 1
ORR	overall response rate
PN	plexiform neurofibroma
PR	partial response
